# Salinosporamide A, a Marine-Derived Proteasome Inhibitor, Inhibits T Cell Activation through Regulating Proliferation and the Cell Cycle

**DOI:** 10.3390/molecules25215031

**Published:** 2020-10-29

**Authors:** Hyun-Su Lee, Gil-Saeng Jeong

**Affiliations:** College of Pharmacy, Keimyung University, 1095 Dalgubeol-daero, Daegu 42601, Korea; hyunsu.lee@kmu.ac.kr

**Keywords:** cell cycle, proteasome inhibitor, salinosporamide A, marine life-derived, T cell proliferation

## Abstract

The appropriate regulation of T cell activity under inflammatory conditions is crucial for maintaining immune homeostasis. Salinosporamide A discovered as a self-resistance product from the marine bacterium *Salinospora tropica*, has been used as a potent proteasome inhibitor (PI). Although PIs have been developed as novel therapeutics for autoimmune diseases, due to their immunosuppressive effect, whether salinosporamide A inhibits T cell activation remains unknown. The current study finds that salinosporamide A is not cytotoxic, but controls T cell proliferation. Results from our cell cycle arrest analysis revealed that salinosporamide A leads to cell cycle arrest and regulates the expression of cyclin-dependent kinases. Under activated conditions, salinosporamide A abrogated T cell activation by T cell receptor-mediated stimulation, in which the production of cytokines was inhibited by pretreatment with salinosporamide A. Furthermore, we demonstrated that the regulation of T cell activation by salinosporamide A is mediated by suppressing the MAPK pathway. Therefore, our results suggest that salinosporamide A effectively suppresses T cell activation through regulating T cell proliferation and the cell cycle and provides great insight into the development of novel therapeutics for autoimmune diseases or graft-versus-host disease.

## 1. Introduction

During the immune response, the proper regulation of T cells is pivotal for maintaining immunological homeostasis, because T cells link humoral immunity to adaptive immunity [1]. Therefore, several events related to T cell activation, including antigenic priming by antigen-presenting cells, activation, and differentiation into effector T cells, should be accurately controlled. Mounting evidence has demonstrated that excessive T cell activity causes a severe immune imbalance or an autoimmune response [2,3]. To control the immunological diseases induced by excessive T cell activation, immunosuppressive reagents have been used. Such agents are categorized into two groups: Inhibitors of pro-inflammatory cytokine production from activated T cells or apoptosis promoters for hazardous T cells.

T cell proliferation is crucial for elevating the adaptive immune response, which is induced by T cell receptor (TCR)-mediated stimulation by co-stimulatory molecules [4,5]. T cell proliferation is initiated by entry into the cell cycle, which is strictly controlled by sequential changes in cyclin expression [6]. During the G1 phase, cyclin D is expressed as the first cyclin protein, while cyclin A is expressed at the entry to the S phase [7]. Previously, it has been found that IL-2, a marker of early T cell activation, acts as the most potent growth factor for T cells [8]. Reduced T cell growth rates have been observed in the presence of neutralizing antibodies against IL-2, and T cells that are stimulated by antigenic engagement or mitogen activation have also shown proliferation upon treatment with IL-2 [9,10]. Several immunosuppressive reagents inhibit T cell proliferation in the presence of TCR-mediated stimulation or lead to cell cycle arrest [11,12]. Although various such reagents have been commercially developed, little is known about whether small molecules derived from natural products regulate T cell activation through proliferation pathways or cell cycle arrest.

**Figure 1:**
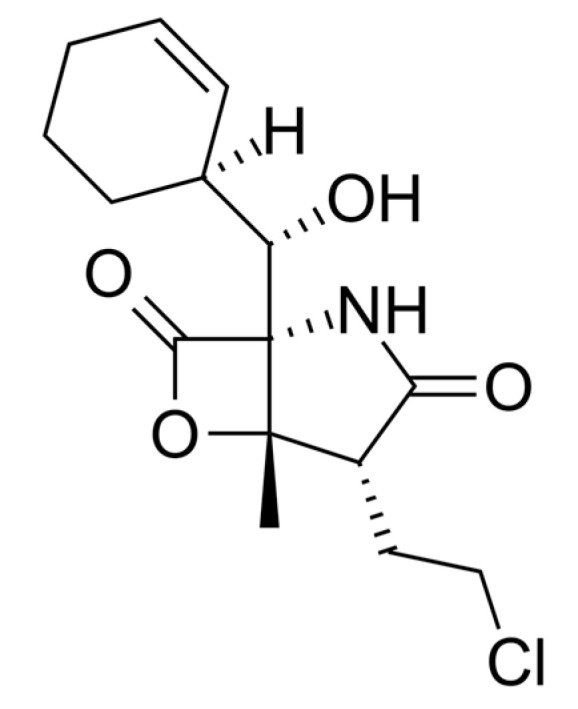
The chemical structure of salinosporamide A.

Salinosporamide A, derived from marine bacteria, such as *Salinospora tropica* and *Salinospora arenicola*, is a potent proteasome inhibitor that was first discovered and structurally elucidated in 2003 [13] (Figure 1). Salinosporamide A has been shown to promote the apoptotic pathway in cancer cells at 35 nM (11 ng/mL) by covalently altering the active site (threonine) of the β–subunit of the 20S proteasome [14]. Although various proteasome inhibitors have been found to inhibit cell proliferation by inducing cell cycle arrest, whether salinosporamide A has a regulatory effect on T cell activation has not yet been reported [15,16]. In the present study, we first found that treatment of T cells with salinosporamide A efficiently suppressed cell proliferation and promoted cell cycle arrest, in a dose-dependent manner. These inhibitory mechanisms result in the inhibition of T cell activity and the MAPK signaling pathway, following stimulation by anti-CD3 and CD28 antibodies.

## 2. Result

### 2.1. High Concentrations of Salinosporamide are Cytotoxic to T Cells and Leads to Apoptosis

**Figure 2:**
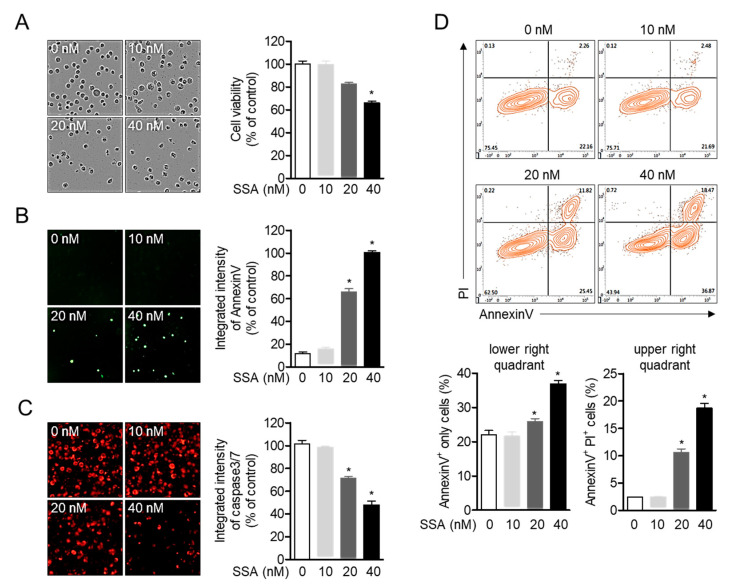
High concentrations of salinosporamide A are cytotoxic to T cells and leads to apoptosis. (**A**–**C**) DIC images (**A**), the expression of AnnexinV (**B**), or caspase3/7 (**C**) in Jurkat T cells obtained from the IncuCyte imaging system after incubation with the indicated concentration of salinosporamide A (0–40 nM) for 24 h. (**D**) The apoptotic population was identified by an AnnexinV/PI apoptosis assay, after incubation with the indicated concentration of salinosporamide A (0–40 nM). The mean value of three experiments ± SEM is presented. * *p* < 0.05 between control cells.

Since salinosporamide A has been reported to exhibit cytotoxicity in HCT-116 colon carcinoma at an IC_50_ of 11 ng/mL (35 nM), we first confirmed whether salinosporamide A is cytotoxic in Jurkat T cells in the presence of up to 40 nM of salinosporamide A. Treatment with salinosporamide A induced cellular death in a dose-dependent manner (<40 nM) (Figure 2A). To determine whether salinosporamide A promotes apoptosis in Jurkat T cells, AnnexinV and caspase3/7 expression levels were evaluated. As shown in Figure 2B,C, AnnexinV expression was significantly upregulated, but caspase3/7 expression was reduced in a dose-dependent manner. The results from the AnnexinV/propidium iodide apoptotic assay confirmed that high concentrations of salinosporamide A (up to 40 nM) lead to Jurkat T cell apoptosis. These data suggest that treatment with high concentrations of salinosporamide A (20 nM and 40 nM) is cytotoxic and induces apoptosis in Jurkat T cells, and this finding is highly consistent with that of a previous report [13].

### 2.2. Treatment with 10 nM Salinosporamide A is not Cytotoxic to T Cells

**Figure 3:**
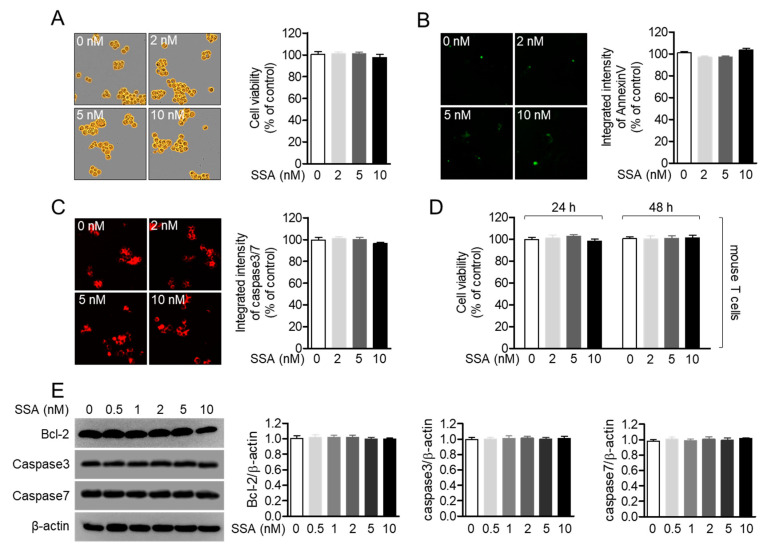
Treatment with 10 nM salinosporamide A is not cytotoxic to T cells. (**A**–**C**) DIC images (**A**), the expression of AnnexinV (**B**), or caspase3/7 (**C**) in Jurkat T cells obtained from the IncuCyte imaging system after incubation with the indicated concentration of salinosporamide A (0–10 nM) for 24 h. (**D**) Mouse CD4^+^ T cells were treated with the indicated concentration of salinosporamide A (0–10 nM) for 24 h or 48 h. After incubation, cell viability was measured by MTT assay. (**E**) Expression levels of the indicated proteins were detected by western blot analysis of Jurkat T cells treated with the indicated concentration of salinosporamide A (0–10 nM) for 24 h. The mean value of three experiments ± SEM is presented.

Despite the fact that salinosporamide A is cytotoxic at concentrations of 20 nM and 40 nM, which has been reported as the IC_50_ value of salinosporamide A, we evaluated whether salinosporamide A was also cytotoxic to Jurkat T cells at concentrations lower than 20 nM. As shown in Figure 3A, Jurkat T cells incubated with 0.5 to 10 nM salinosporamide A did not exhibit cell death after 24 h. The expression levels of AnnexinV and caspase3/7 in Jurkat T cells treated with up to 10 nM salinosporamide A also demonstrated that salinosporamide A does not cause Jurkat T cell apoptosis at these concentrations (Figure 3B,C). To address whether treatment with salinosporamide A affects primary T cells isolated from spleen and lymph nodes, MTT assay was performed after treatment with 2, 5, 10 nM salinosporamide A of mouse T cells. Figure 3D revealed that treatment with salinosporamide A was not cytotoxic to primary T cells up to 10 nM salinosporamide A. Furthermore, the expression levels of anti-apoptotic proteins, including Bcl-2, Caspase3, and Caspase7, did not change in the presence of salinosporamide A, after treatment for 24 h (Figure 3E). These data indicate that treatment with up to 10 nM salinosporamide A does not promote cell death and apoptosis in Jurkat T cells and mouse T cells.

### 2.3. Treatment with 10 nM Salinosporamide A Reduces T Cell Proliferation

**Figure 4:**
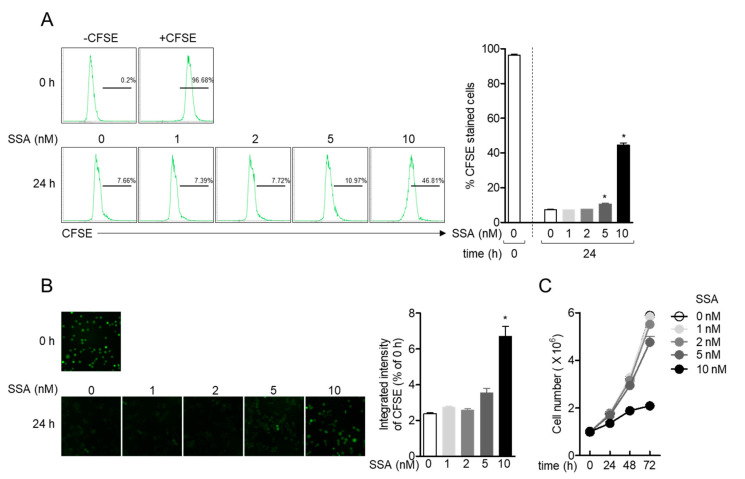
Treatment with 10 nM salinosporamide A reduces T cell proliferation. (**A**, **B**) Jurkat T cells pre-stained with 1 μM CFSE for 30 min were treated with the indicated concentration of salinosporamide A (0–10 nM) for 24. The percentage of CFSE-positive cells were measured by flow cytometry (**A**), and microscopic fluorescence images and integrated CFSE intensity were determined using an IncuCyte imaging system (**B**). The black line refers to CFSE-positive cells according to the negative control (-CFSE). (**C**) The growth rate of Jurkat T cells treated with the indicated concentration of salinosporamide (0–10 nM) for 72 h was determined by counting the cell number every 24 h. The mean value of three experiments ± SEM is presented. * *p* < 0.05 between control cells.

To elucidate whether treatment with 10 nM salinosporamide A affects T cell proliferation, a carboxyfluorescein succinimidyl ester (CFSE) proliferation assay was performed with Jurkat T cells incubated with 10 nM salinosporamide A, by flow cytometry and IncuCyte imaging system. The suppressive effect of salinosporamide A on Jurkat T cell proliferation was determined by flow cytometry (Figure 4A), and suppression of CFSE-positive Jurkat T cells treated with salinosporamide A after 24 h was significantly downregulated, in a dose-dependent manner. Obtained microscopic images by IncuCyte imaging system also confirmed that treatment with salinosporamide A of Jurkat T cells reduced the attenuation of CFSE intensity after 24 h incubation compared to control cells (Figure 4B). Furthermore, the growth rate of Jurkat T cells were observed in the presence of salinosporamide A within 72 h. Figure 4C revealed that treatment with 10 nM salinosporamide A dramatically inhibits the growth rate of Jurkat T cells. These data suggest that salinosporamide A attenuates T cell proliferation, at concentrations of up to 10 nM.

### 2.4. Treatment with 10 nM Salinosporamide A Leads to Cell Cycle Arrest and Regulates Cyclin-Dependent Kinase Expression in T Cells

**Figure 5:**
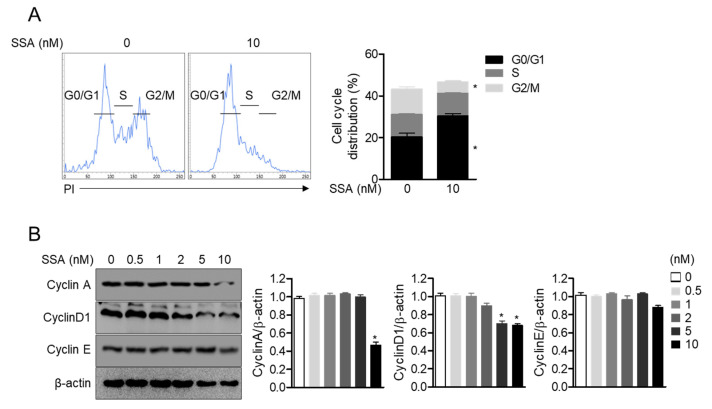
Treatment with 10 nM salinosporamide A leads to cell cycle arrest and regulates cyclin-dependent kinase expression in T cells. (**A**) Cell cycle distribution of T cells treated with 10 nM salinosporamide A for 24 h was determined by a PI staining assay using flow cytometry. (**B**) Expression levels of the indicated proteins were detected by western blot analysis of Jurkat T cells treated with the indicated concentration of salinosporamide A (0–10 nM) for 24 h. The mean value of three experiments ± SEM is presented. * *p* < 0.05 between control cells.

T cell proliferation is tightly controlled by the expression of cyclins that regulate the cell cycle [17]. To evaluate the mechanism by which salinosporamide A dampens T cell proliferation, the effect of this molecule on the cell cycle was determined. The result obtained from the cell cycle assay showed that the entry into G2/M phase in the cell cycle was significantly blocked by treatment with salinosporamide A. To confirm whether treatment with salinosporamide A affects the cyclin proteins, the expression levels of cyclinA, cyclinD1, and cyclinE were detected by western blotting. The expression of cyclinA and cyclinD1 was significantly downregulated by treatment with salinosporamide A, but comparable cyclinE expression was observed in the presence of salinosporamide A (Figure 5B). These data indicate that treatment with salinosporamide A, at concentrations of up to 10 nM, efficiently arrests the cell cycle by blocking G2/M phase entry in Jurkat T cells.

### 2.5. Treatment with 10 nM Salinosporamide A Inhibits T-Cell Activity Following TCR-Mediated and PMA/A23187 Stimulation

**Figure 6:**
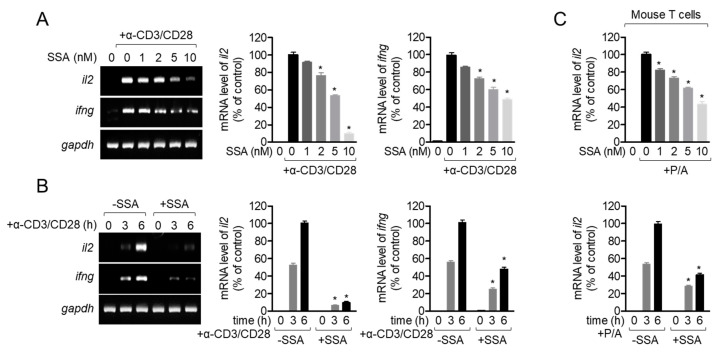
Treatment with 10 nM salinosporamide A inhibits T-cell activity following TCR-mediated and PMA/A23187 stimulation. (**A**) Jurkat T cells pre-treated with the indicated concentration of salinosporamide A (0–10 nM) for 1 h were stimulated with anti-CD3 (20 μg/mL)/CD28 (7 μg/mL) antibodies for 6 h. mRNA levels of *il2* and *ifng* were measured by conventional and quantitative PCR. (**B**) Jurkat T cells pre-treated with the 10 nM salinosporamide A for 1 h were stimulated with anti-CD3 (20 μg/mL)/CD28 (7 μg/mL) antibodies for the indicated time (0–6 h). mRNA levels of *il2* and *ifng* were measured by conventional and quantitative PCR. (**C**) Mouse T cells pre-treated with the indicated concentration of salinosporamide A (0–10 nM) or 10 nM salinosporamide A for 1 h were stimulated with PMA (100 nM)/A23187 (1 μM) for 6 h or indicated time (0–6 h). After cells were harvested, the mRNA level of *il2* was determined by quantitative PCR. The mean value of three experiments ± SEM is presented. * *p* < 0.05 between control cells.

Accumulating evidence has shown that T cell activity and pro-inflammatory cytokine production are highly associated with proliferation and the cell cycle. To elucidate whether incubation with salinosporamide A reduces T cell activity, the mRNA levels of *il2* and *ifng* were measured in Jurkat T cells that were activated with anti-CD3/CD28 antibodies. Figure 6A shows that pretreatment with salinosporamide A reduces pro-inflammatory cytokine mRNA levels in a dose-dependent manner. In particular, treatment with from 2 nM of salinosporamide A reveals a suppressive effect on the expression of pro-inflammatory cytokines. The inhibition of T cell pro-inflammatory cytokine production by salinosporamide A was confirmed by time-dependent experiments (Figure 6B). To address whether salinosporamide A attenuates the expression of pro-inflammatory cytokines, *il2* mRNA levels were detected from mouse T cells stimulated with PMA/A23187. Figure 6C revealed that pretreatment with salinosporamide A significantly downregulates mRNA level of *il2* from stimulated mouse T cells with PMA/A23187 in a dose and time-dependent manner. These data suggest that treatment with salinosporamide A reduces the production of pro-inflammatory cytokines by Jurkat T cells and mouse T cells.

### 2.6. Treatment with 10 nM Salinosporamide A Dampens the MAPK Pathway in Activated T Cells

**Figure 7:**
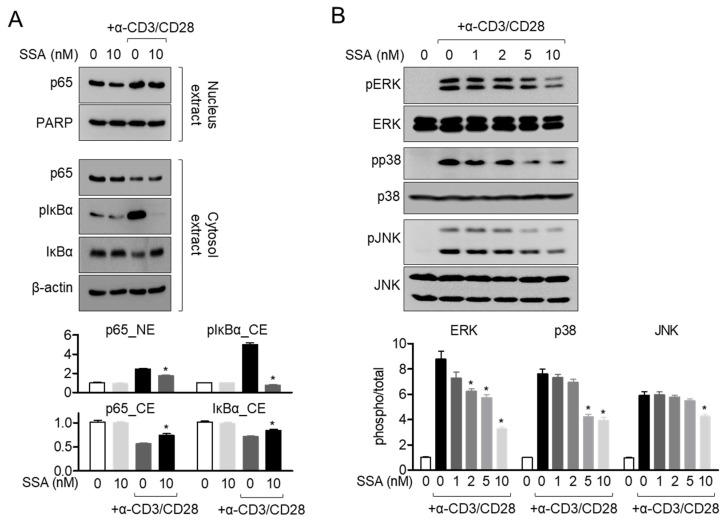
Treatment with 10 nM salinosporamide A dampens the MAPK pathway in activated T cells. (**A**) Nuclear-translocated p65 and phosphorylated or degraded IκBα from activated Jurkat T cells pre-treated with 10 nM salinosporamide A were detected by western blot analysis. Separation of nucleus extract from the whole lysates was performed using the NE-PER kit. Nuclear protein was normalized to the expression of PARP, and cytosol protein was normalized to the expression of β-actin. (**B**) The phosphorylation levels of ERK, p38, and JNK from activated Jurkat T cells pre-treated with the indicated concentration of salinosporamide A were detected by western blot analysis. All phosphorylation levels were normalized to the total levels of each respective protein and presented in a bar graph. The mean value of three experiments ± SEM is presented. * *p* < 0.05 between control cells.

NFκB and MAPK have been elucidated as the major signaling pathways that promote T cell activation in Jurkat T cells [18]. To check whether pretreatment with salinosporamide A suppresses T cell activation through the NFκB and MAPK pathways, we first examined the translocation of p65 in activated T cells pretreated with salinosporamide A. Figure 7A shows that p65 translocation into the nucleus is partially blocked in Jurkat T cells pretreated with salinosporamide A. Phosphorylation and degradation of IκBα were also observed (Figure 7A). To assess whether pretreatment with salinosporamide A influences the MAPK pathway in activated T cells, the phosphorylation levels of MAPK signaling molecules, including ERK, p38, and JNK, were detected by western blotting. Figure 7B shows that pretreatment with salinosporamide A efficiently suppressed the phosphorylation of MAPK signaling molecules, in a dose-dependent manner. These data indicate that treatment with up to 10 nM salinosporamide inhibits T cell activation through suppressing NFκB and MAPK pathways in activated T cells.

## 3. Discussion

In the present study, we demonstrated that salinosporamide A derived from marine life suppresses T cell activation by regulating T cell proliferation and entry into the cell cycle. First, it was clearly confirmed that salinosporamide A was not cytotoxic at concentrations below 10 nM in Jurkat T cells and mouse T cells. Furthermore, it has been reported that treatment with salinosporamide A leads to apoptosis at a higher concentration of 35 nM. Treatment with 10 nM salinosporamide A regulated the ability of Jurkat T cells to proliferate and enter into the G2/M phase of the cell cycle. The abrogated proliferation and cell cycle entry that were observed upon treatment with salinosporamide A were associated with a reduction in the production of pro-inflammatory cytokines, including IL-2 and IFNγ, by T cells in the presence of TCR-mediated stimulation of the NFκB and MAPK pathways.

T cells proliferation and entry into the cell cycle are pivotal processes that occur after antigenic engagement with antigen-presenting cells in the early phase of inflammatory conditions [19]. Upon TCR-mediated stimulation, T cell proliferation and cell cycle entry are tightly controlled by several regulatory proteins that undergo proteasomal degradation [20,21]. A recent report has demonstrated that the activity of the proteasome is highly involved in the differentiation of CD8^+^ T cells into effector and memory T cells [22]. Due to the influence of proteasome inhibitors in regulating various T cell processes, finding the ‘optimal concentration’ that is effective without showing cytotoxicity is important in the pharmacological approach. In the current study, we first validated whether salinosporamide A leads to cytotoxicity in T cells, in a dose-dependent manner, and evaluated its pharmacological effects on T cell proliferation and entry into the cell cycle at a concentration of 10 nM. Although we observed that 40 nM of salinosporamide A induces cellular apoptosis by performing an apoptosis assay [13], we also found that 10 nM of salinosporamide A inhibits T cell activation by abrogating proliferation and entry into the cell cycle. Further studies should be conducted to determine whether 10 nM salinosporamide A or less regulates the functions of T cells, such as differentiation into effector T cells or supervision of immune cells without cytotoxicity.

Several proteasome inhibitors have been developed as immunosuppressive reagents, due to their regulatory roles in antigen processing and presentation and in signaling cascades that regulate cellular functions and survival [23]. The biological functions of proteasome inhibitors have been recently identified in immune and cancer cells, and most proteasome inhibitors have been found to attenuate NFκB signaling [24,25]. A previous study has also evaluated a promising proteasome inhibitor as an immunosuppressive reagent in animal and clinical experiments and investigated transplant rejection using specific blocking antibodies and graft versus host disease [26]. Another trial investigated whether bortezomib attenuates autoimmune disorders, including rheumatoid arthritis and Sjögren’s syndrome [27]. Interestingly, bortezomib has been shown to downregulate activated T cell proliferation and IL-2 production, similar to the findings of salinosporamide A in the current study [28,29]. We found that pretreatment with salinosporamide A inhibited T cell activation by repressing T cell proliferation and cell cycle entry. These results suggest that salinosporamide A can be developed as an immunosuppressive reagent to treat autoimmune disorders and transplant rejection.

## 4. Materials and Methods

### 4.1. Cells

Jurkat T cells were obtained from the Korean cell line bank (Seoul, Korea). Cells were cultured in RPMI medium (Welgene, Gyeongsan, Korea) supplemented with penicillin G (100 units/mL) and streptomycin (100 μg/mL), 10% fetal bovine serum (FBS), and L-glutamine (2 mM) at 37 °C in a humidified incubator containing 5% CO_2_ and 95% air.

### 4.2. Reagents and Antibodies

AnnexinV and caspase3/7 staining reagents for IncuCyte^®^ cell imaging system were obtained from Essen bio (Ann Arbor, MI, USA). AnnexinV/PI apoptosis assay kit was purchased from BD Biosciences (San Diego, CA, USA). Anti-CD3 and anti-CD28 for stimulation were obtained from Bioxcell (West Lebanon, NH, USA). Salinosporamide A, MTT (1-(4,5-Dimethylthiazol-2-yl)-3,5-diphenylformazan) powder, PMA (Phorbol 12-myristate 13-acetate), and A23187 was purchased from Sigma Chemical Co. (St. Louis, MO, USA). Carboxyfluorescein succinimidyl ester (CFSE) dye, ECL Western blotting detection reagents, and NE-PER Nuclear and Cytoplasmic Extraction Reagents Kit were obtained from Thermo Fisher Scientific (Waltham, MA, USA). PVDF membrane was obtained from Bio-Rad (Hercules, CA, USA). Anti-bcl-2, anti-β-actin, anti-cyclin A, anti-cyclin D1, and cyclin E antibodies were purchased from Santa Cruz Biotechnology (Dallas, TX, USA). Anti-caspase3, anti-caspase7, anti-p65, anti-PARP, anti-IκBα, anti-ERK, anti-p38, anti-JNK, anti-phosphorylated IκBα (S32), anti-phosphorylated ERK (T202/Y204), anti-phosphorylated p38 (T180/Y182), and anti-phosphorylated JNK (T183/Y185) antibodies were obtained from cell signaling technology (Danvers, MA, USA).

### 4.3. MTT Assay

Cell viability was assessed by MTT assay. Jurkat T cells or mouse T cells (1 × 10^4^ cells/well, 96 wells) were incubated with indicated concentrations (0–40 nM) of salinosporamide A for 24 h at 37 °C. After incubation, MTT (500 μg/mL) was added to the incubated cells for 2 h, and the supernatant was discarded for dissolving formazan crystal by adding 150 μL of DMSO to each well. Plates were read at 540 nm, and the cell viability was obtained by calculating normalized absorbance of each sample with absorbance from control cells. Cell viability was presented in % of control.

### 4.4. Assessment of the Expressions of AnnexinV and Caspase3/7 by IncuCyte

The expressions of AnnexinV or caspase3/7 after incubation of salinosporamide A were assessed by IncuCyte imaging system (Sartorius, Ann Arbor, MI, USA). Jurkat T cells (1 × 10^4^ cells/well, 96 wells) were seeded and incubated with indicated concentrations of salinosporamide (0–40 nM) for 24 h. Before incubation, reagents for staining AnnexinV (1 μM, green) and caspase3/7 (1 μM, red) were added to the cell medium. Fluorescence from cells was acquired by IncuCyte imaging system, and the integrated intensity of AnnexinV or caspase3/7 were automatically calculated.

### 4.5. AnnexinV/PI Apoptosis Assay

The apoptotic population was evaluated by AnnexinV/PI apoptosis assay. Jurkat cells (1 × 10^5^ cells/well, 24 wells) treated with the indicated concentration of salinosporamide A (0–40 nM) for 24 h were stained with AnnexinV and PI following manufacture’s instruction. AnnexinV/PI-double positive population was presented in a bar graph.

### 4.6. Isolation of Mouse T Cells

Naïve C57BL/6 mice were sacrificed, and CD4^+^ T cells were isolated from the LNs and spleens by magnetic-activated cell sorting (MACS) separation (Miltenyi Biotec, Bergisch Gladbach, Germany). All experiments were approved by the Animal Care and Use Committee of the College of Pharmacy, Keimyung University (approval number: KM2020-007).

### 4.7. Western Blot Assay

Western blot assay was performed to detect the expression in protein level as a reference [30]. Jurkat cells were lysed in RIPA buffer for 30 min on ice and centrifuged at 14,000 rpm for 20 min at 4 °C. For separation of nuclear extract from the whole lysate, cells were lysed with NE-PER Nuclear and Cytoplasmic Extraction Reagents (Thermo Fisher Scientific, Waltham, MA, USA) following the manufacturer’s instructions. Approximately 30 μg of the lysate was loaded on 8–12% SDS–PAGE gels for separation. Proteins were transferred on PVDF membranes (Bio-Rad, Hercules, CA, USA). Membranes were blocked with 5% skim milk for 1 h, rinsed with TBS containing 0.1% Tween 20 (TBS-T). Membranes were incubated with the indicated primary antibodies in 3% skim milk in TBS-T overnight at 4 °C. The next day, excess primary antibodies were removed with TBS-T, and then membranes were incubated with 0.1 μg/mL peroxidase-labeled secondary antibodies (against rabbit or mouse) for 1 h. After three washes with TBS-T, bands were visualized with ECL Western blotting detection reagents (Thermo Fisher Scientific, Waltham, MA, USA) with an ImageQuant LAS 4000 (GE healthcare, Chicago, IL, USA). Representative images from three independent experiments are shown in figures, and all detected bands were normalized to β–actin for whole lysate or cytosol extracts and PARP for nucleus extracts.

### 4.8. Proliferation Assay

Jurkat T cells (1 × 10^5^ cells/well, 24 wells) pre-stained with 1 μM CFSE for 30 min at 37 °C were treated with the indicated concentration (0–10 nM) for 24 h. For obtaining images, cells were acquired by IncuCyte imaging system, and integrated intensity of CFSE from each well were automatically calculated. Cells were also acquired by flow cytometry for the determination of % CFSE stained cells.

### 4.9. Cell Cycle Arrest Assay

Jurkat T cells (1 × 10^5^ cells/well, 24 wells) were incubated with 10 nM salinosporamide A for 24 h and harvested for PI staining. Before staining with PI, cells were fixed with 70% cold ethanol for 1 h. After three times wash with 1X cold PBS, cells were stained with 10 μg/mL PI for 30 min. Stained cells were acquired by flow cytometry, and histogram observed were separated in G0/G1, S, and G2/M phase. Cell cycle distribution was presented in the % G0/G1, S, and G2/M phase.

### 4.10. Real-Time Quantitative PCR

To measure mRNA levels of genes from cells, total RNA was isolated using TRIZOL reagent (JBI, Korea), and reverse transcription was performed using RT PreMix. Primers used for each gene were as follows (forward and reverse primers, respectively): human *il2*, 5′-CAC GTC TTG CAC TTG TCA C-3′ and 5′-CCT TCT TGG GCA TGT AAA ACT-3′; human *ifng*, 5′-TGA CCA GAG CAT CCA AAA GA-3′ and 5′-CTC TTC GAC CTC GAA ACA GC-3′; human *gapdh*, 5′-CGG AGT CAA CGG ATT TGG TCG TAT-3′ and 5′-AGC CTT CTC CAT GGT GGT GAA GAC-3′; mouse *il2*, 5′-TGA GCA GGA TGG AGA ATT ACA GG-3′ and 5′-GTC CAA GTT CAT CTT CTA GGC AC-3′; mouse *gapdh*, 5′-CGG AGT CAA CGG ATT TGG TCG TAT-3′ and 5′-AGC CTT CTC CAT GGT GGT GAA GAC-3′. PCR conditions were as follows: Forty cycles of denaturation at 94 °C for 30 s, annealing at 60 °C for 20 s, and extension at 72 °C for 40 s; followed by denaturation at 72 °C for 7 min. For quantitative real-time PCR analysis, DNA Engine Opticon 1 continuous fluorescence detection system (MJ Research, Waltham, MA, USA) with SYBR Premix Ex Taq (Takara, Japan) was used. The total reaction volume was 10 μL. It contained 1 μL of cDNA/control and gene-specific primers. Each PCR reaction was performed using the following conditions: 94 °C 30 s, 60 °C 30 s, 72 °C 30 s, and the plate read (detection of fluorescent product) for 40 cycles followed by 7 min of extension at 72 °C. Melting curve analysis was performed to characterize the dsDNA product by slowly increasing the temperature (0.2 °C/s) from 65 °C to 95 °C with fluorescence data collected at 0.2 °C intervals. mRNA levels of inflammatory cytokines normalized to *gapdh* were expressed as fold changes relative to those of untreated controls. The fold change in gene expression was calculated using the following equation: Fold change = 2 − ΔΔCT, where ΔΔCT = (CT Target – CT *gapdh*) at time x – (CT Target – CT *gapdh*) at time 0 h. Here, time x represents any time point, and time 0 represents the 1 X expression of the target gene in the untreated cells normalized to *gapdh*.

### 4.11. Statistics

For statistical analysis, mean values ± SEM were calculated from the result of three independent experiments performed and presented in graphs. A one-way ANOVA was used to obtain significance (P value). * indicates differences between indicated groups considered significant at *p* < 0.05.
